# Supplementary material for: The pro-inflammatory effect of Staphylokinase contributes to community-associated Staphylococcus aureus pneumonia
Source: Commun Biol. 2022 Jun 23;5:618. doi: 10.1038/s42003-022-03571-x (PMC9226170; doi:10.1038/s42003-022-03571-x)

The pro-inflammatory effect of Staphylokinase contributes to community-associated Staphylococcus aureus pneumonia

Yanan Wang[1#], Na Zhao[1#], Ying Jian[1], Yao Liu[1], Lin Zhao[1], Lei He[1], Qian Liu[1]*, Min Li[1,2]*

1. Department of Laboratory Medicine, Ren Ji Hospital, Shanghai Jiao Tong University School of Medicine, Shanghai 200127, China.

2. Faculty of Medical Laboratory Science, College of Health Science and Technology, Shanghai Jiao Tong University School of Medicine, Shanghai 200025, China.

# These authors contributed equally to this work.

*** Corresponding author:**

Min Li. Email: ruth_limin@126.com

Qian Liu. Email: qq2005011@163.com

Supplementary Information: File contains supplementary methods, supplementary figures and figure legends, supplementary tables and unprocessed western blot images.

Supplementary Methods

Bacterial isolates and growth conditions

The definitions of CA-SA and HA-SA refer to a previously published paper[1]. Unless otherwise stated, *Escherichia coli* was routinely grown in Luria-Bertani medium, and *S. aureus* was grown in tryptic soy broth (TSB) (Oxoid) or agar plates. TSB medium was also used when measuring the growth of *S. aureus*, and the absorbance of the bacterial suspension at 600_{nm} wavelength was measured every hour.

Molecular typing

Multi-locus sequence typing (MLST) of *S. aureus* isolates was performed by detection of seven housekeeping genes (*arcC*, *aroE*, *glpF*, *gmk*, *pta*, *tpi*, and *yqiL*)[2]. Sequences of these housekeeping genes were submitted to the *S. aureus* MLST database for comparison (https://pubmlst.org/).

Allelic gene replacement by homologous recombination and genetic complementation

We selected a representative ST398 clinical isolate from respiratory specimens to delete the *sak* gene. The homologous recombination procedure was performed by using plasmid *pKOR1* as described[3]. The *sak* complementary plasmid was generated by cloning the *sak* gene into the vector *pOS1*.

Abreviation list of cytokines and genes in Figure 3

G-CSF: Granulocyte Colony Stimulating Factor

GM-CSF: Granulocyte-macrophage Colony Stimulating Factor

KC: keratinocyte chemokine

MCP-1: macrophage chemoattractant protein-1

MIP: macrophage inflammatory protein

RANTES: regulated upon activation normal T cell expressed and secreted factor

Eotaxin: Eosinophil Chemotactic Protein

Casp1/8: Caspase 1/8

Tlr2/6/7/8/9/12: toll-like receptor 2/6/7/8/9/12

Tirap: toll-interleukin 1 receptor domain-containing adaptor protein

Mapk3: mitogen-activated protein kinase 3

Nfkb1/2: nuclear factor of kappa light polypeptide gene enhancer in B cells 1/2

Myd88: myeloid differentiation primary response gene 88

Irak1/4: interleukin-1 receptor-associated kinase 1/4

Irf5/7: interferon regulatory factor 5/7

Ripk1: receptor-interacting serine-threonine kinase 1

Mlkl: mixed lineage kinase domain-like

Supplementary Figures

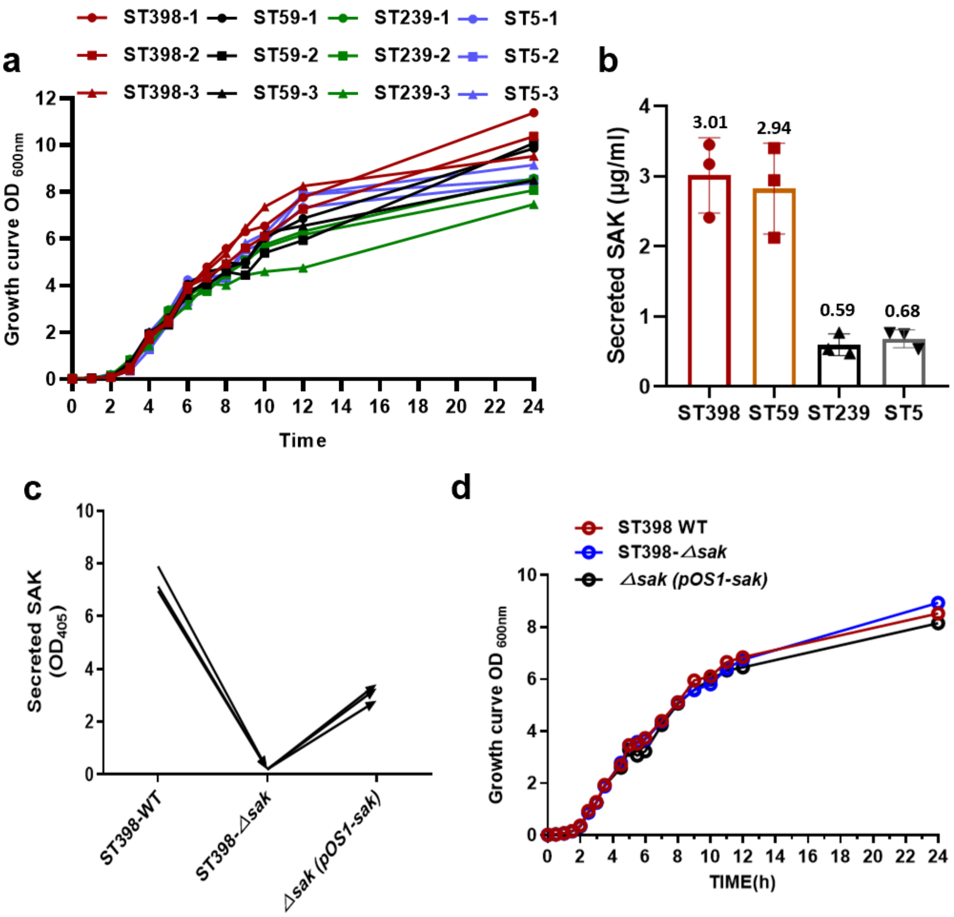

Supplementary Figure 1. The deletion of *sak* did not affect the growth of ST398. a Growth curve of *S. aureus* clinical isolates. **b** SAK levels secreted by *S. aureus* in culture supernatants (stationary phase). Compare the absorbance of the test sample with the absorbance value of the recombinant SAK standard dilution to estimate the SAK content in the sample. **c** Measurement of SAK secretion to confirm the successful construction of *sak* gene knockout and complemented strains. **d** Growth of the *sak* deletion and complemented mutants was comparable to that of the wild-type strain.

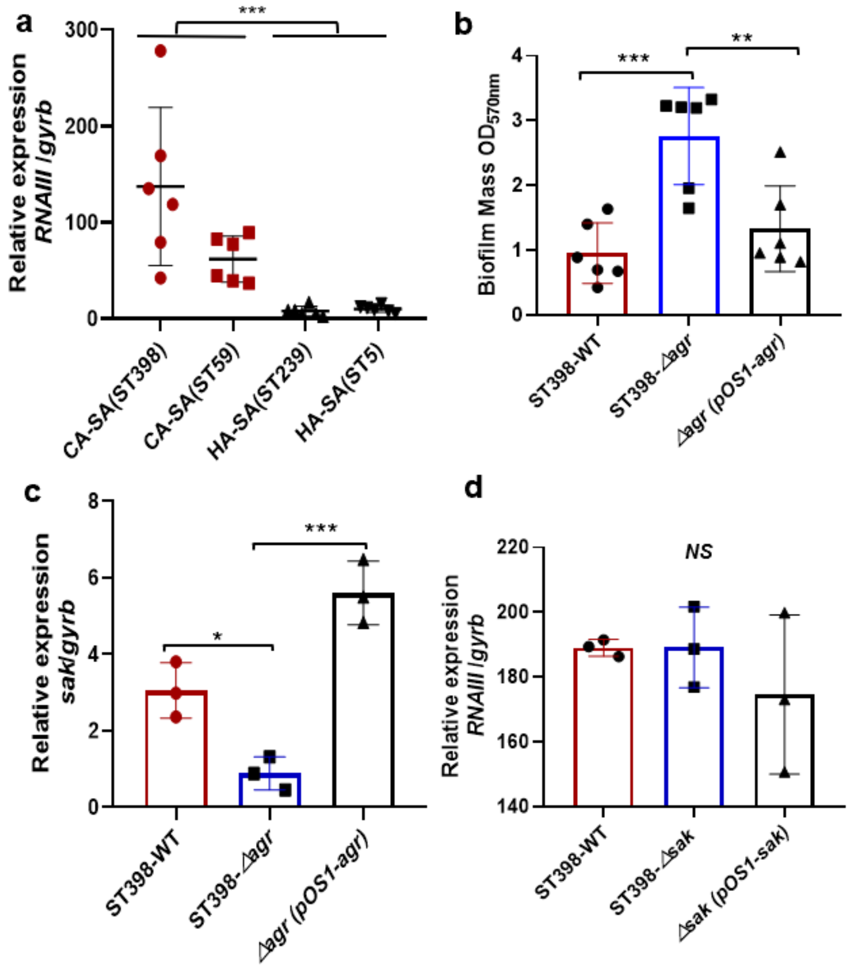

Supplementary Figure 2. The expression level of *agr* and its effect on biofilm formation and *sak* expression. a qRT-PCR analysis of *RNAIII* gene expression in randomly selected clinical CA-SA ST398, CA-SA ST59, HA-SA ST239, and HA-SA ST5 isolates, at 4 h of *in-vitro* growth. Relative mRNA levels were calculated using *gyrb* as control and expressed as 2^(-ΔΔCt). Unpaired *t* test was used for statistical analyses between CA-SA and HA-SA. **b** The effect of Agr on the biofilm formation of ST398 isolate and unpaired *t* test was used for statistical analyses. **c, d** qRT-PCR analysis of *sak* or *RNAIII* gene expression. Relative mRNA levels were calculated using *gyrb* as control and expressed as 2^(-ΔΔCt). Unpaired *t* test was used for statistical analyses after Shapiro–Wilk normality test. All data are presented as mean ± SD and *$p<0.05$, ** $p<0.01$, *** $p<0.001$.

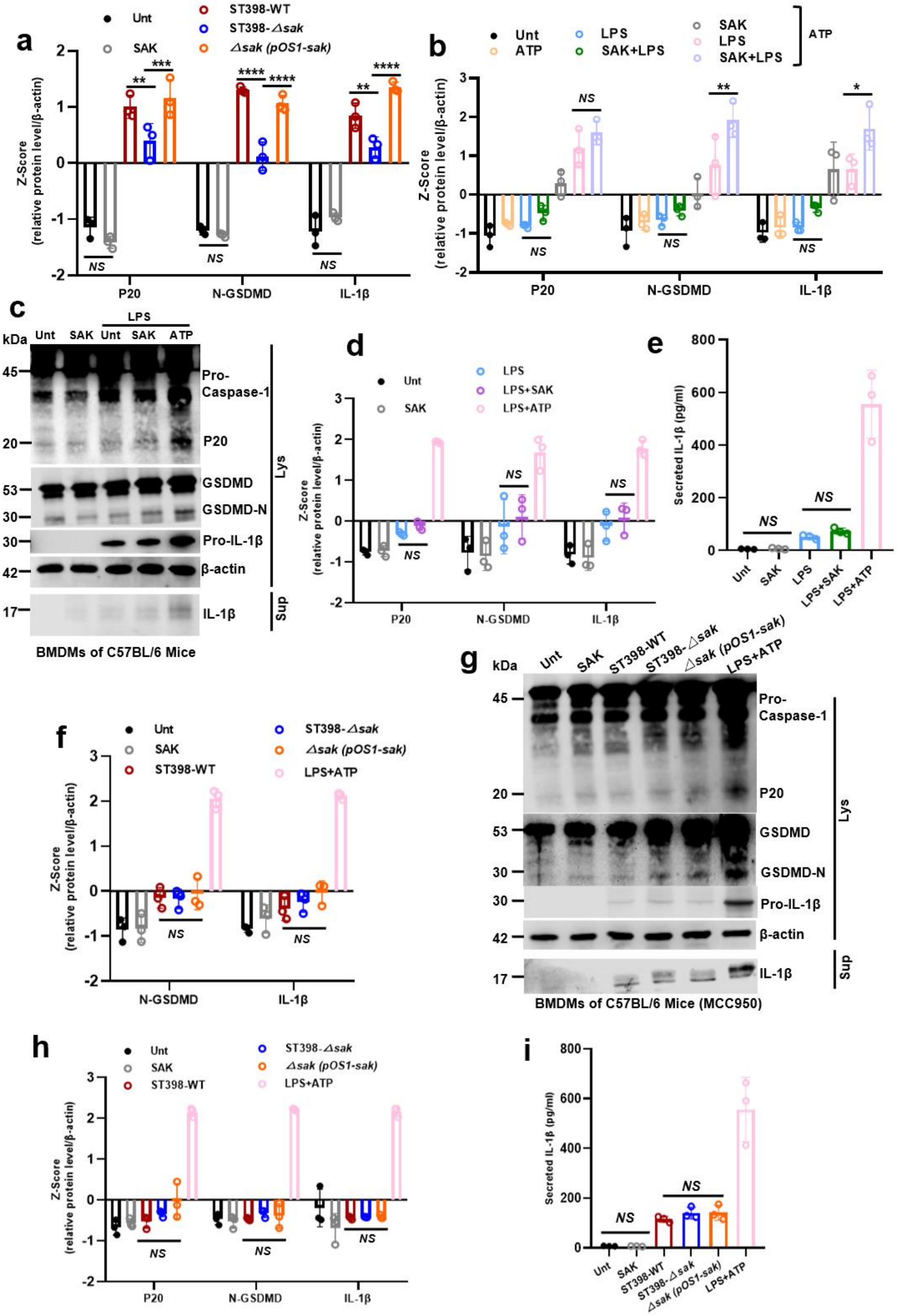

Supplementary Figure 3. Role of SAK in promoting the activation of NLRP3 inflammasome. a, b The protein expression levels were detected by western blot and analyzed by relative densitometric quantification (the data were normalized by the Z score according to the standard deviation from the mean). **c-e** BMDMs were incubated with LPS (0.2μg/ml) for 3 hours, followed by the addition of SAK (0.5μg/ml) or ATP

(2.5mM) for 30 minutes. Western blot analysis of cell lysates or culture supernatants of treated mouse BMDMs (**c**) and quantification of the densitometry (the data were normalized by the Z score according to the standard deviation from the mean) of each band (**d**). Secreted IL-1β from BMDMs was detected by ELISA (**e**). **f** Quantification of protein expression by immunoblot analysis of cell lysates or culture supernatants of BMDMs from *Caspase-1*$^{-/-}$ mice (the data were normalized by the Z score according to the standard deviation from the mean). **g-i** BMDMs were pretreated with MCC950 (1μM) and then incubated with SAK (0.5μg/ml) or bacterial secretion supernatant for 3 hours (stationary phase, 1:20 dilution). Cell lysates or culture supernatants of mouse BMDMs treated with LPS (0.2μg/ml, 3h) and ATP (2.5mM, 30min) were used as controls. Western blot analysis of cell lysates or culture supernatants of treated mouse BMDMs (**g**) and quantification of the densitometry (the data were normalized by the Z score according to the standard deviation from the mean) of each band (**h**). Secreted IL-1β from BMDMs was detected by ELISA (**i**). Two-way ANOVA with Bonferroni's multiple comparison post-test was used to compare the differences between the variables. All data are presented as mean ± SD and *p<0.05, ** p<0.01, *** p<0.001.

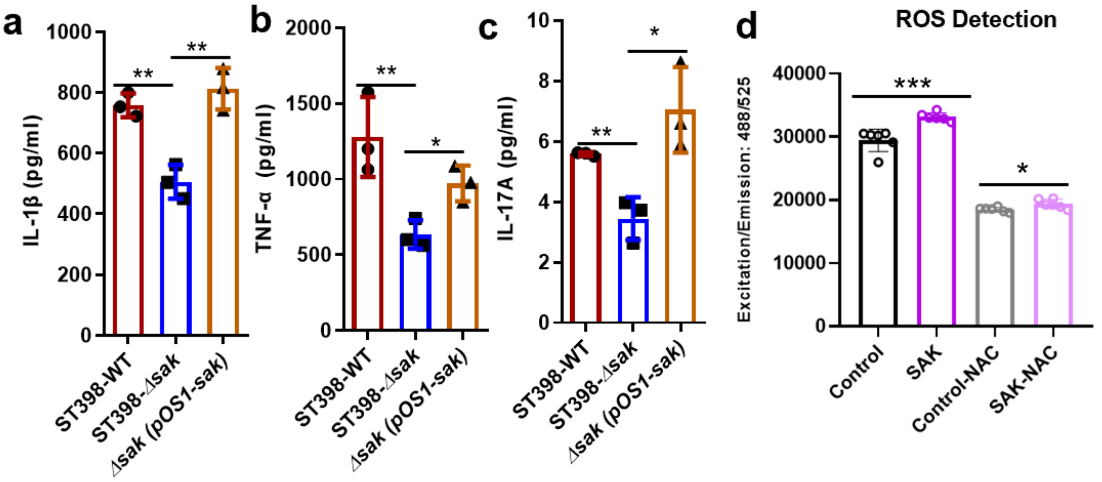

Supplementary Figure 4. SAK can stimulate the release of inflammatory factors in THP-1 cells and promote the production of ROS. a-c Cultured THP1 cells (human monocyte derived cell line) were incubated with bacterial secretion supernatant. The physiologically relevant concentrations of specific cytokine proteins in THP1 cell culture supernatants were detected by ELISA kit. **d** Reactive Oxygen Species Assay. THP1 cells were pretreated with NAC (1mM) and then incubated with SAK (0.5μg/ml) for 3 hours. Unpaired t-test was used to compare the differences between the variables after Shapiro–Wilk normality test. All data are presented as mean ± SD and *p<0.05, ** p<0.01, *** p<0.001.

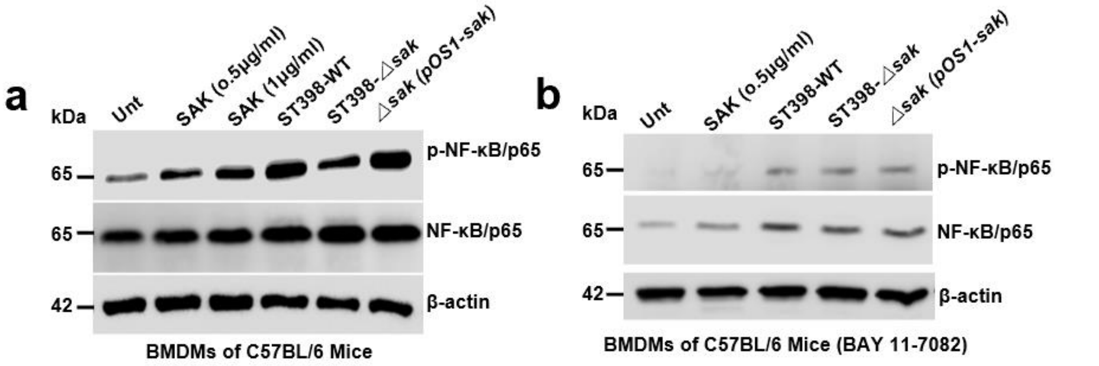

Supplementary Figure 5. SAK can promote the activation of NF-κB. a Immunoblot analysis of cell lysates from mouse BMDMs treated with SAK (0.5μg/ml, 1μg/ml, 3h) or bacterial supernatants (stationary phase, 1:20 dilution, 3h). **b** Mouse BMDMs were pretreated with BAY 11-7082 (10 μM, 30 min), then cells were treated with SAK (0.5 μg/ml) or bacterial secretory supernatant for 3 hours (stationary phase, 1:20 dilution). Cell lysates were used for western blot analysis.

Supplementary Tables

Supplementary Table 1. Bacterial strains and plasmids used in this study.

Strains/plasmids	Relevant genotype and property	Source/reference
S. aureus		
RN4220	derived from NCTC8325-4; r-m+	Ref:[4]
ST398	CA-SA clinical isolate	This study
ST398-*△sak*	ST398 *sak* mutant	This study
△sak (pOS1-sak)	ST398 *sak* mutant with *pOS1-sak*	This study
ST398-*△agr*	ST398 *agr* mutant	This study
△agr (pOS1-agr)	ST398 *agr* mutant with *pOS1-agr*	This study
E. coli		

DH5α	*endA1 recA1 gyrA96 thi-1 hsdR17(rK-mK+) relA1 supE44 (lacZYA-argF) U169 F-80dlacZM15 deoR phoA*	Invitrogen
**Plasmids**		
pKOR1	*cmR* and *ampR*, temperature sensitive vector for allelic replacement via lambda recombination and *ccdB* selection	Ref:[3]
pOS1	*E. coli*/Staphylococcus shuttle cloning plasmid, *cmR*, *ampR*	Ref:[5]

138

139 **Supplementary Table 2. Oligonucleotides used in this study.**

Oligonucleotide	Sequence
**Oligonucleotides for isogenic deletion mutants**	
sak-att1	GGGGACAAGTTTGTACAAAAAAGCAGGCTGCAATTAACAGACGGTTTAAACG
sak-rev1	GGCGCTTCCTCCAAATATAATATA
sak-rev2	TTATATTTGGAGGAAGCGCCTAGTTGTTTATTATAGAAAGCAA
sak-att2	GGGGACCACTTTGTACAAGAAAGCTGGGTTCTTTTTTATATAAAGGTTTG
agr-att1	GGGGACAAGTTTGTACAAAAAAGCAGGCTACCCTTTCAATTGTCTGACG

agr-rev1	GATGAATAATTAATTACTTTCATTGTAAA
agr-rev2	AGTAATTAATTATTCATCACTTACCTATTTAACGTTTGT CTACA
agr-att2	GGGGACCACTTTGTACAAGAAAGCTGGGTGGGATGCC TTTATTGGTG

Oligonucleotides for genetic complementation

sak-Sam1-F	GAGCCCGGGAAGCGCCATGCTCAAAAGAGGT
sak-Bamh1-his-R	GAGGGATCCTTAatgatgatgatgatgatgTTTCTTTTCTATAATA ACC

Oligonucleotides for qRT- PCR

sak-F	GAGGTAAGTGCATCAAGTTC
sak-R	GACATAATGAGGGGATAGCA
gyrB-F	CAAATGATCACAGCATTTGGTACAG
gyrB-R	CGGCATCAGTCATAATGACGAT
RNAIII-F	ATAGCACTGAGTCCAAGGAAACTAACT
RNAIII-R	GCCATCCCAACTTAATAACCATGT
NLRP3-mice-F	GGGAGACCGTGAGGAAAGGA
NLRP3-mice-R	CCAAAGAGGAATCGGACAACAAA
caspase-1-mice-F	CAGGCAAGCCAAATCTTTATCACT
caspase-1-mice-R	GTGCCATCTTCTTTGTTCTGTTCTT
β-actin-mice-F	TGAGAGGGAAATCGTGCGTGAC
β-actin-mice-R	GCTCGTTGCCAATAGTGATGACC

IFN-α-mice-F	GAGAGCCTTGACACTCCTGG
IFN-α-mice-R	GCTTGAGCCTTCTTGATCTGC
TNF-α-mice-F	CAGGCGGTGCCTATGTCTC
TNF-α-mice-R	CGATCACCCCGAAGTTCAGTAG
IL-17A-mice-F	GTGTCTCTGATGCTGTTG
IL-17A-mice-R	AACGGTTGAGGTAGTCTG
IL-1β-mice-F	CAGGCAGGCAGTATCACTCA
IL-1β-mice-R	AGCTCATATGGGTCCGACAT

Supplementary References

1 Wang, Y. *et al.* Role of the ESAT-6 secretion system in virulence of the emerging community-associated Staphylococcus aureus lineage ST398. *Sci Rep* **6**, 25163, doi:10.1038/srep25163 (2016).

2 Ji, Y. Methicillin-resistant Staphylococcus aureus (MRSA) protocols. Preface. *Methods Mol Biol* **391**, v, doi:10.1007/978-1-59745-468-1 (2007).

3 Bae, T. & Schneewind, O. Allelic replacement in Staphylococcus aureus with inducible counter-selection. *Plasmid* **55**, 58-63, doi:10.1016/j.plasmid.2005.05.005 (2006).

4 Azavedo, J., Foster, T. J., Hartigan, P. J., Arbuthnott, J. P. & Novick, R. P. Expression of the cloned toxic shock syndrome toxin 1 gene (tst) in vivo with a rabbit uterine model. *Infection & Immunity* **50**, 304 (1985).

5 Wardenburg, J. B., Williams, W. A. & Missiakas, D. Host defenses against Staphylococcus aureus infection require recognition of bacterial lipoproteins. *Proceedings of the National Academy of Sciences* **103**, 13831-13836 (2006).

Unprocessed western blot images

Figure 4g

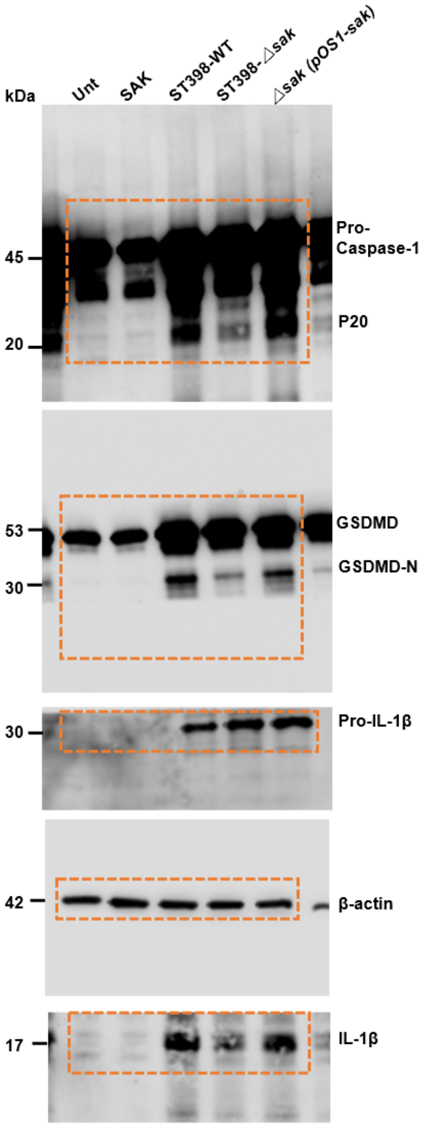

Figure 4i

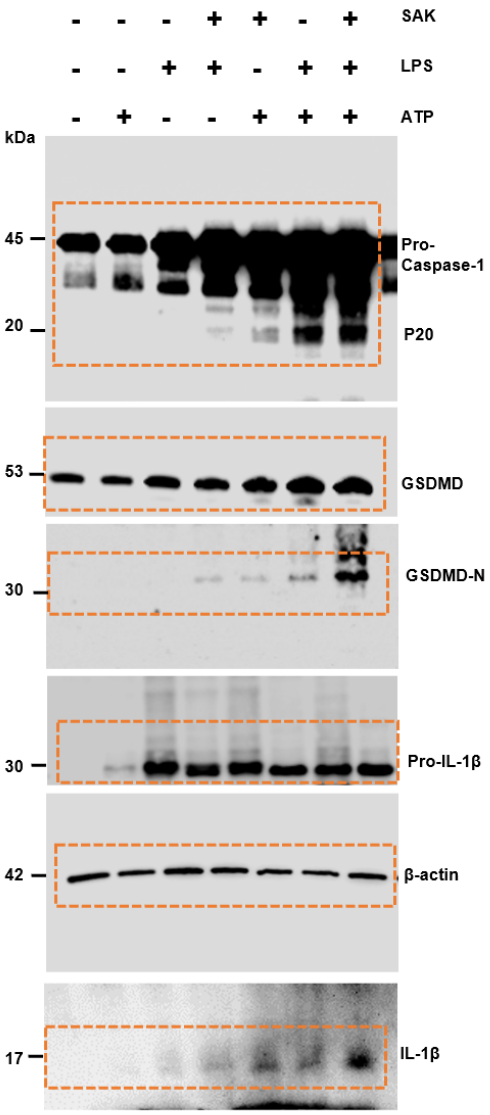

Figure 4k

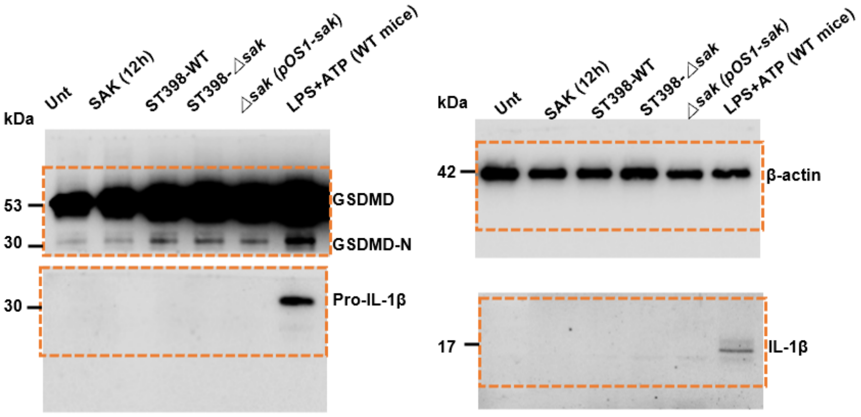

Supplementary Figure 3c

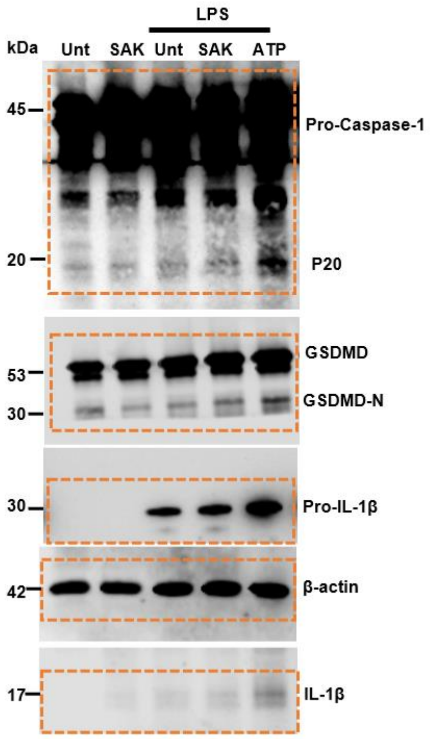

Supplementary Figure 3g

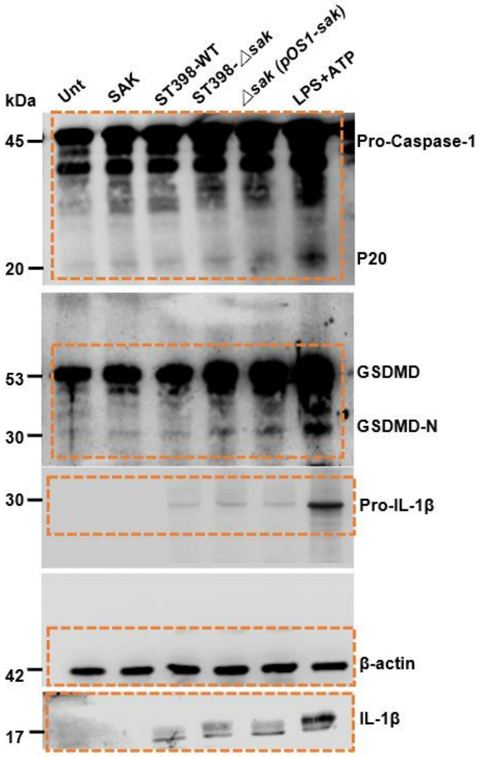

Supplementary Figure 5a

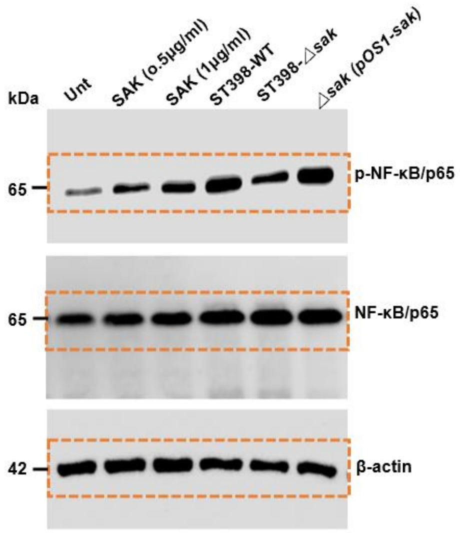

Supplementary Figure 5b

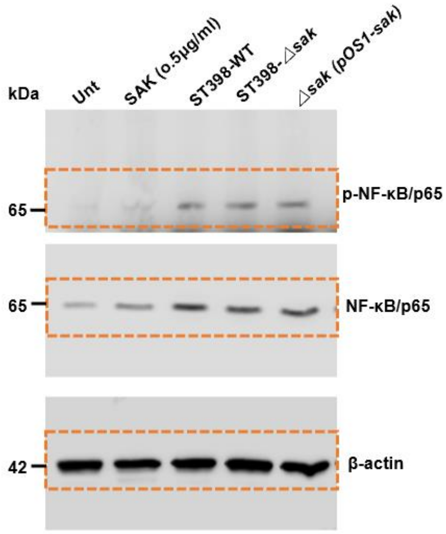

Supplement: Supplementary file 2 — Supplementary Information FINAL [file 42003_2022_3571_MOESM2_ESM.pdf]
