# Supplementary material for: The pro-inflammatory effect of Staphylokinase contributes to community-associated Staphylococcus aureus pneumonia
Source: Commun Biol. 2022 Jun 23;5:618. doi: 10.1038/s42003-022-03571-x (PMC9226170; doi:10.1038/s42003-022-03571-x)
Supplement: Supplementary file 3 — Description of Supplementary Files [file 42003_2022_3571_MOESM3_ESM.docx]

**Description of Additional Supplementary Files**

**File name:** Supplementary Data 1

**Description:** Top 50 significantly different genes in RNA-seq analysis of mouse lung tissue.

**File name:** Supplementary Data 2

**Description:** Contains all normalized data for main figures and supplementary figures.
